# (2*E*)-4-*tert*-Butyl-2-(4-meth­oxy­benzyl­idene)-3,4-dihydro­naphthalen-1(2*H*)-one

**DOI:** 10.1107/S1600536811021969

**Published:** 2011-06-25

**Authors:** Mohamed Akhazzane, Hafid Zouihri, Maria Daoudi, Abdelali Kerbal, Ghali Al Houari

**Affiliations:** a Laboratoire de Chimie Organique, Faculté des Sciences Dhar el Mahraz, Université Sidi Mohammed Ben Abdellah, Fès, Morocco; b Laboratoire de Diffraction des Rayons X, Centre National pour la Recherche Scientifique et Technique, Rabat, Morocco

## Abstract

In the title compound C_22_H_24_O_2_, the exocyclic C=C double bond is in an *E* configuration and the *tert*-butyl group is in an axial position on the cyclo­hexa­none ring. The cyclo­hexa­none ring in the dihydro­naphthalene fused-ring system adopts a half-chair conformation in both independent two mol­ecules in the asymetric unit. The benzene ring system is oriented angles of 43.97 (12) and 39.24 (12)° with respect to the naphthyl ring system in the two independent mol­ecules. In the crystal, mol­ecules are linked *via* C—H⋯O hydrogen bonds and C—H⋯π inter­actions.

## Related literature

For general background to dipolar 1,3-cyclo­addition reactions, see: Bennani *et al.* (2007 ▶); Kerbal *et al.* (1988 ▶); Al Houari *et al.* (2008 ▶). For a related structure, see: Akhazzane *et al.* (2010 ▶). For conformation analysis, see: Cremer & Pople (1975 ▶).
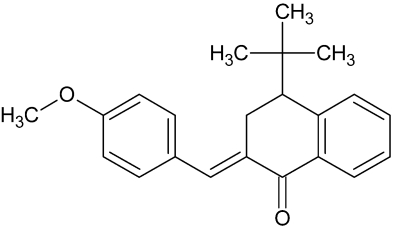


## Experimental

### 

#### Crystal data

C_22_H_24_O_2_
*M* *_r_* = 320.41
Orthorhombic, 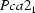

*a* = 11.2265 (2) Å
*b* = 21.7630 (5) Å
*c* = 14.8001 (4) Å
*V* = 3615.99 (14) Å^3^
*Z* = 8
Mo *K*α radiation
μ = 0.07 mm^−1^
*T* = 296 K
0.24 × 0.17 × 0.16 mm

#### Data collection

Bruker APEXII CCD detector diffractometer
20114 measured reflections
6654 independent reflections
4552 reflections with *I* > 2σ(*I*)
*R*_int_ = 0.033

#### Refinement

*R*[*F* ^2^ > 2σ(*F* ^2^)] = 0.056
*wR*(*F* ^2^) = 0.147
*S* = 1.03
6654 reflections
441 parameters
1 restraint
H-atom parameters constrained
Δρ_max_ = 0.41 e Å^−3^
Δρ_min_ = −0.21 e Å^−3^

### 

Data collection: *APEX2* (Bruker, 2005 ▶); cell refinement: *SAINT* (Bruker, 2005 ▶); data reduction: *SAINT*; program(s) used to solve structure: *SHELXS97* (Sheldrick, 2008 ▶); program(s) used to refine structure: *SHELXL97* (Sheldrick, 2008 ▶); molecular graphics: *PLATON* (Spek, 2009 ▶); software used to prepare material for publication: *publCIF* (Westrip, 2010 ▶).

## Supplementary Material

Crystal structure: contains datablock(s) I, New_Global_Publ_Block. DOI: 10.1107/S1600536811021969/dn2694sup1.cif

Structure factors: contains datablock(s) I. DOI: 10.1107/S1600536811021969/dn2694Isup2.hkl

Supplementary material file. DOI: 10.1107/S1600536811021969/dn2694Isup3.cml

Additional supplementary materials:  crystallographic information; 3D view; checkCIF report

## Figures and Tables

**Table 1:** Hydrogen-bond geometry (Å, °) *Cg*3 and *Cg*7 are the centroids of the C111–C116 and C211–C216 rings, respectively.

*D*—H⋯*A*	*D*—H	H⋯*A*	*D*⋯*A*	*D*—H⋯*A*
C104—H104⋯O21^i^	0.93	2.53	3.439 (3)	167
C121—H12*D*⋯*Cg*7	0.96	2.92	3.801 (4)	153
C221—H22*F*⋯*Cg*3	0.96	2.86	3.686 (4)	144

Symmetry code: (i) 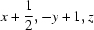.

**Fig. 1.**
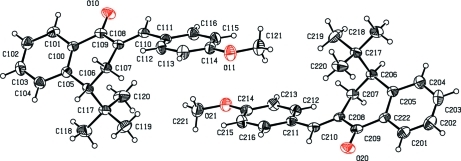
Molecular view of the title compound showing the atom-labeling scheme. Displacement ellipsoids are drawn at the 30% probability level. H atoms are represented as small spheres of arbitrary radii.

## supplementary crystallographic information

### Comment

Knowledge of the configuration and conformation of the title compound is necessary to understand its behaviour in dipolar-1,3 cycloaddition reactions [Bennani, *et al.* (2007) and Al Houari *et al.* (2008)]. To confirm the E configuration of the exocyclic C=C double bond, an X-ray crystal structure determination has been carried out.

In the title compound, as shown in Fig. 1, all bond lengths and angles are normal and comparable with those reported for the related structure [Akhazzane *et al.*, (2010)].

The asymmetric unit cell contains two molecules (Fig.1). The cyclohexanone ring in the dihydronaphthalin ring system has a half-chair conformation, presumably due to conjugation of the planar annulated benzo ring, with the puckering parameters of: Q(2) = 0.419 (3) Å, φ(2) = 148.0 (4)°, Q(3) = 0.189 (3) Å and: Q(2) = 0.410 (3) Å., φ(2) = 89.4 (4) °, Q(3) = 0.192 (3) Å in the two independent molecules, respectivly. (Cremer & Pople, 1975).

In the crystal, molecules are linked *via* C—H···O hydrogen bonds (Table. 1). The crystal structure is further stablized by intermolecular C—H···π interactions between the methyl of the methoxybenzene and the neighboring benzene ring.

### Experimental

The synthesis of 2-(4-methoxybenzylidene)-4-tertiobutyl-3,4-dihydronaphthalen-1(2*H*)-one was achieved using the method reported by [Kerbal *et al.* (1988)]. *i.e.* by a condensation of *para* anisaldehyde with 4-tertiobutyl-3,4-dihydronaphthalen-1(2*H*)-one in an alkaline medium in methanol.

### Refinement

The H atoms bound to C were treated as riding with their parent atoms [C—H distances are 0.93Å for CH groups with *U*_iso_(H) = 1.2 *U*_eq_(C), and 0.97 Å for CH_3_ groups with *U*_iso_(H) = 1.5 *U*_eq_(C)].

### Crystal data

**Table:**

C_22_H_24_O_2_	*F*(000) = 1376
*M**_r_* = 320.41	*D*_x_ = 1.177 Mg m^−^^3^
Orthorhombic, *P**c**a*2_1_	Mo *K*α radiation, λ = 0.71073 Å
Hall symbol: P 2c -2ac	Cell parameters from 256 reflections
*a* = 11.2265 (2) Å	θ = 2.4–25.8°
*b* = 21.7630 (5) Å	µ = 0.07 mm^−^^1^
*c* = 14.8001 (4) Å	*T* = 296 K
*V* = 3615.99 (14) Å^3^	Prism, colourless
*Z* = 8	0.24 × 0.17 × 0.16 mm

### Data collection

**Table:**

Bruker APEXII CCD detector diffractometer	4552 reflections with *I* > 2σ(*I*)
Radiation source: fine-focus sealed tube	*R*_int_ = 0.033
graphite	θ_max_ = 26.5°, θ_min_ = 0.9°
ω and φ scans	*h* = −13→14
20114 measured reflections	*k* = −24→27
6654 independent reflections	*l* = −16→18

### Refinement

**Table:**

Refinement on *F*^2^	Primary atom site location: structure-invariant direct methods
Least-squares matrix: full	Secondary atom site location: difference Fourier map
*R*[*F*^2^ > 2σ(*F*^2^)] = 0.056	Hydrogen site location: inferred from neighbouring sites
*wR*(*F*^2^) = 0.147	H-atom parameters constrained
*S* = 1.03	*w* = 1/[σ^2^(*F*_o_^2^) + (0.088*P*)^2^] where *P* = (*F*_o_^2^ + 2*F*_c_^2^)/3
6654 reflections	(Δ/σ)_max_ = 0.011
441 parameters	Δρ_max_ = 0.41 e Å^−^^3^
1 restraint	Δρ_min_ = −0.21 e Å^−^^3^

### Special details

**Table:**

Geometry. All e.s.d.'s (except the e.s.d. in the dihedral angle between two l.s. planes) are estimated using the full covariance matrix. The cell e.s.d.'s are taken into account individually in the estimation of e.s.d.'s in distances, angles and torsion angles; correlations between e.s.d.'s in cell parameters are only used when they are defined by crystal symmetry. An approximate (isotropic) treatment of cell e.s.d.'s is used for estimating e.s.d.'s involving l.s. planes.
Refinement. Refinement of *F*^2^ against ALL reflections. The weighted *R*-factor *wR* and goodness of fit *S* are based on *F*^2^, conventional *R*-factors *R* are based on *F*, with *F* set to zero for negative *F*^2^. The threshold expression of *F*^2^ > σ(*F*^2^) is used only for calculating *R*-factors(gt) *etc*. and is not relevant to the choice of reflections for refinement. *R*-factors based on *F*^2^ are statistically about twice as large as those based on *F*, and *R*- factors based on ALL data will be even larger.

### Fractional atomic coordinates and isotropic or equivalent isotropic displacement parameters (Å^2^)

**Table:**

	*x*	*y*	*z*	*U*_iso_*/*U*_eq_	
C100	0.6470 (2)	0.38382 (11)	0.4388 (2)	0.0411 (6)	
C101	0.6110 (2)	0.33059 (12)	0.4851 (2)	0.0532 (7)	
C102	0.6531 (3)	0.27392 (13)	0.4598 (3)	0.0622 (8)	
C103	0.7326 (3)	0.26978 (13)	0.3892 (3)	0.0641 (9)	
C104	0.7685 (2)	0.32188 (12)	0.3434 (2)	0.0552 (7)	
C105	0.7248 (2)	0.38008 (11)	0.36673 (19)	0.0424 (6)	
C106	0.7596 (2)	0.43672 (10)	0.31438 (19)	0.0415 (6)	
C107	0.7704 (3)	0.49220 (11)	0.3798 (2)	0.0429 (7)	
C108	0.6628 (2)	0.50071 (10)	0.4383 (2)	0.0403 (7)	
C109	0.5989 (2)	0.44403 (12)	0.4701 (2)	0.0440 (7)	
C110	0.6144 (2)	0.55490 (11)	0.4650 (2)	0.0444 (7)	
C111	0.6550 (2)	0.61823 (12)	0.4470 (2)	0.0439 (7)	
C112	0.7729 (2)	0.63626 (11)	0.4354 (2)	0.0534 (8)	
C113	0.8008 (2)	0.69646 (12)	0.4200 (2)	0.0605 (8)	
C114	0.7139 (3)	0.74091 (12)	0.4167 (2)	0.0531 (8)	
C115	0.5966 (3)	0.72479 (12)	0.4296 (2)	0.0577 (8)	
C116	0.5687 (3)	0.66397 (12)	0.4458 (2)	0.0583 (8)	
C117	0.6807 (2)	0.44838 (12)	0.22824 (19)	0.0475 (7)	
C118	0.6908 (3)	0.39284 (16)	0.1651 (2)	0.0806 (10)	
C119	0.7281 (4)	0.50413 (15)	0.1770 (3)	0.0739 (12)	
C120	0.5498 (2)	0.45874 (18)	0.2510 (3)	0.0786 (11)	
C121	0.6667 (3)	0.84831 (13)	0.4022 (3)	0.0801 (11)	
C201	0.6361 (3)	1.16663 (12)	0.0896 (2)	0.0603 (8)	
C202	0.5944 (3)	1.22365 (14)	0.1102 (3)	0.0714 (10)	
C203	0.5132 (3)	1.23039 (13)	0.1791 (3)	0.0699 (11)	
C204	0.4739 (2)	1.17962 (12)	0.2283 (2)	0.0579 (8)	
C205	0.5184 (2)	1.12162 (11)	0.20951 (19)	0.0426 (6)	
C206	0.4829 (2)	1.06589 (11)	0.26467 (19)	0.0407 (6)	
C207	0.4753 (3)	1.00980 (13)	0.2027 (2)	0.0438 (7)	
C208	0.5855 (2)	0.99897 (10)	0.1451 (2)	0.0378 (7)	
C209	0.6456 (2)	1.05429 (12)	0.1115 (2)	0.0437 (7)	
C210	0.6308 (2)	0.94469 (11)	0.1232 (2)	0.0442 (7)	
C211	0.5944 (2)	0.88216 (12)	0.1428 (2)	0.0428 (7)	
C212	0.4772 (2)	0.86312 (12)	0.1611 (2)	0.0476 (7)	
C213	0.4502 (2)	0.80294 (11)	0.1798 (2)	0.0504 (7)	
C214	0.5383 (3)	0.75867 (12)	0.1790 (2)	0.0496 (8)	
C215	0.6544 (2)	0.77552 (12)	0.1599 (2)	0.0589 (8)	
C216	0.6790 (2)	0.83588 (12)	0.1417 (2)	0.0547 (7)	
C217	0.5618 (2)	1.05718 (11)	0.3510 (2)	0.0452 (6)	
C218	0.5489 (3)	1.11228 (14)	0.4134 (2)	0.0682 (9)	
C219	0.5204 (5)	1.00059 (14)	0.4031 (3)	0.0789 (13)	
C220	0.6944 (2)	1.04974 (16)	0.3289 (2)	0.0761 (11)	
C221	0.5906 (3)	0.65256 (12)	0.1973 (3)	0.0718 (9)	
C222	0.5994 (2)	1.11478 (12)	0.1365 (2)	0.0406 (7)	
H101	0.5583	0.3338	0.5333	0.064*	
H102	0.6281	0.2387	0.4900	0.075*	
H103	0.7624	0.2316	0.3722	0.077*	
H104	0.8228	0.3183	0.2962	0.066*	
H106	0.8403	0.4290	0.2919	0.050*	
H10A	0.8396	0.4863	0.4181	0.052*	
H10B	0.7831	0.5293	0.3448	0.052*	
H110	0.5456	0.5517	0.4998	0.053*	
H112	0.8333	0.6071	0.4381	0.064*	
H113	0.8800	0.7076	0.4116	0.073*	
H115	0.5370	0.7544	0.4274	0.069*	
H116	0.4897	0.6533	0.4563	0.070*	
H11A	0.8092	0.4968	0.1594	0.111*	
H11B	0.7245	0.5397	0.2152	0.111*	
H11C	0.6804	0.5109	0.1241	0.111*	
H11D	0.6521	0.4019	0.1088	0.121*	
H11E	0.6532	0.3579	0.1926	0.121*	
H11F	0.7733	0.3838	0.1543	0.121*	
H12A	0.5066	0.4679	0.1967	0.118*	
H12B	0.5428	0.4925	0.2924	0.118*	
H12C	0.5177	0.4223	0.2783	0.118*	
H12D	0.6148	0.8442	0.3510	0.120*	
H12E	0.7072	0.8871	0.3992	0.120*	
H12F	0.6207	0.8463	0.4568	0.120*	
H201	0.6908	1.1623	0.0428	0.072*	
H202	0.6205	1.2578	0.0779	0.086*	
H203	0.4843	1.2693	0.1931	0.084*	
H204	0.4176	1.1847	0.2739	0.070*	
H206	0.4018	1.0737	0.2862	0.049*	
H20A	0.4615	0.9736	0.2395	0.053*	
H20B	0.4071	1.0147	0.1631	0.053*	
H210	0.6996	0.9472	0.0884	0.053*	
H212	0.4163	0.8921	0.1606	0.057*	
H213	0.3721	0.7919	0.1931	0.060*	
H215	0.7148	0.7463	0.1595	0.071*	
H216	0.7572	0.8464	0.1277	0.066*	
H21A	0.5970	1.1062	0.4662	0.102*	
H21B	0.4669	1.1167	0.4309	0.102*	
H21C	0.5744	1.1487	0.3824	0.102*	
H21D	0.5608	0.9988	0.4602	0.118*	
H21E	0.5382	0.9643	0.3688	0.118*	
H21F	0.4360	1.0031	0.4131	0.118*	
H22A	0.7213	1.0847	0.2950	0.114*	
H22B	0.7059	1.0131	0.2937	0.114*	
H22C	0.7391	1.0467	0.3840	0.114*	
H22D	0.6287	0.6507	0.1394	0.108*	
H22E	0.5534	0.6138	0.2101	0.108*	
H22F	0.6489	0.6614	0.2430	0.108*	
O10	0.51340 (18)	0.44643 (8)	0.52145 (17)	0.0632 (6)	
O11	0.7524 (2)	0.79948 (8)	0.40158 (18)	0.0742 (7)	
O20	0.73570 (19)	1.05053 (8)	0.06354 (16)	0.0657 (6)	
O21	0.50231 (18)	0.69992 (8)	0.19669 (16)	0.0639 (6)	

### Atomic displacement parameters (Å^2^)

**Table:**

	*U*^11^	*U*^22^	*U*^33^	*U*^12^	*U*^13^	*U*^23^
C100	0.0373 (14)	0.0491 (15)	0.0369 (16)	−0.0045 (12)	−0.0024 (13)	0.0011 (13)
C101	0.0571 (17)	0.0589 (17)	0.0436 (18)	−0.0105 (13)	0.0010 (14)	0.0046 (14)
C102	0.074 (2)	0.0477 (16)	0.065 (2)	−0.0026 (14)	0.0001 (17)	0.0072 (15)
C103	0.070 (2)	0.0445 (15)	0.078 (3)	0.0091 (16)	−0.0053 (19)	−0.0052 (16)
C104	0.0481 (16)	0.0600 (16)	0.058 (2)	0.0076 (13)	−0.0010 (14)	−0.0029 (15)
C105	0.0349 (14)	0.0490 (13)	0.0433 (17)	0.0012 (11)	−0.0073 (12)	−0.0024 (12)
C106	0.0301 (12)	0.0516 (14)	0.0429 (16)	0.0020 (11)	0.0038 (11)	−0.0047 (12)
C107	0.0359 (15)	0.0509 (14)	0.0420 (19)	−0.0005 (11)	0.0009 (13)	−0.0037 (13)
C108	0.0369 (17)	0.0494 (18)	0.0347 (18)	−0.0027 (10)	0.0020 (16)	−0.0018 (10)
C109	0.0373 (16)	0.0582 (17)	0.0366 (18)	−0.0070 (12)	0.0006 (14)	−0.0034 (13)
C110	0.0425 (16)	0.0555 (17)	0.0353 (18)	0.0003 (12)	0.0009 (13)	−0.0046 (13)
C111	0.0478 (17)	0.0484 (15)	0.0356 (17)	0.0019 (13)	0.0002 (14)	−0.0067 (13)
C112	0.0429 (16)	0.0490 (15)	0.068 (2)	0.0053 (12)	−0.0141 (14)	−0.0067 (14)
C113	0.0430 (16)	0.0589 (17)	0.079 (2)	−0.0047 (13)	−0.0091 (15)	−0.0010 (16)
C114	0.057 (2)	0.0472 (15)	0.056 (2)	0.0006 (13)	−0.0072 (15)	0.0006 (14)
C115	0.0543 (18)	0.0510 (16)	0.068 (2)	0.0108 (13)	0.0060 (15)	0.0006 (15)
C116	0.0471 (17)	0.0599 (17)	0.068 (2)	0.0043 (13)	0.0112 (15)	0.0004 (16)
C117	0.0410 (14)	0.0669 (16)	0.0345 (15)	0.0031 (12)	0.0009 (11)	−0.0042 (13)
C118	0.100 (3)	0.094 (2)	0.049 (2)	0.008 (2)	−0.0109 (19)	−0.0183 (19)
C119	0.070 (3)	0.097 (3)	0.055 (3)	0.0033 (16)	−0.002 (2)	0.0214 (18)
C120	0.0443 (17)	0.143 (3)	0.049 (2)	0.0093 (18)	−0.0089 (15)	0.005 (2)
C121	0.089 (2)	0.0523 (17)	0.099 (3)	0.0078 (16)	−0.002 (2)	0.0062 (18)
C201	0.0626 (19)	0.0606 (17)	0.057 (2)	−0.0112 (15)	0.0004 (16)	0.0115 (16)
C202	0.078 (2)	0.0596 (19)	0.077 (3)	−0.0132 (16)	−0.007 (2)	0.0219 (18)
C203	0.069 (2)	0.0457 (17)	0.095 (3)	0.0081 (16)	−0.015 (2)	0.0045 (18)
C204	0.0490 (16)	0.0574 (17)	0.067 (2)	0.0017 (13)	−0.0032 (14)	−0.0039 (15)
C205	0.0347 (13)	0.0511 (14)	0.0419 (17)	−0.0028 (11)	−0.0076 (11)	0.0005 (13)
C206	0.0302 (12)	0.0524 (15)	0.0396 (16)	−0.0021 (10)	0.0037 (11)	−0.0001 (12)
C207	0.0363 (16)	0.0539 (14)	0.0412 (18)	−0.0112 (11)	0.0025 (13)	−0.0006 (14)
C208	0.0363 (17)	0.0478 (18)	0.0292 (17)	−0.0069 (10)	−0.0042 (15)	−0.0009 (11)
C209	0.0442 (17)	0.0560 (17)	0.0307 (16)	−0.0095 (13)	−0.0025 (14)	−0.0017 (12)
C210	0.0385 (14)	0.0583 (17)	0.0357 (18)	−0.0052 (13)	0.0012 (12)	0.0000 (13)
C211	0.0386 (15)	0.0543 (16)	0.0356 (17)	−0.0007 (12)	−0.0044 (13)	−0.0004 (13)
C212	0.0398 (14)	0.0554 (15)	0.0475 (17)	0.0015 (11)	−0.0014 (12)	−0.0041 (13)
C213	0.0381 (14)	0.0539 (16)	0.059 (2)	−0.0064 (12)	0.0039 (12)	−0.0030 (14)
C214	0.0522 (19)	0.0525 (16)	0.0440 (19)	−0.0039 (13)	−0.0036 (14)	−0.0060 (13)
C215	0.0524 (18)	0.0563 (16)	0.068 (2)	0.0093 (14)	−0.0005 (15)	−0.0010 (16)
C216	0.0412 (15)	0.0617 (17)	0.061 (2)	0.0008 (13)	0.0061 (14)	0.0001 (15)
C217	0.0412 (14)	0.0563 (15)	0.0381 (16)	0.0012 (11)	0.0046 (12)	−0.0027 (12)
C218	0.081 (2)	0.0767 (19)	0.047 (2)	0.0087 (17)	−0.0085 (17)	−0.0109 (16)
C219	0.113 (3)	0.068 (2)	0.055 (3)	−0.0074 (17)	−0.014 (2)	0.0202 (17)
C220	0.0406 (16)	0.140 (3)	0.047 (2)	0.0144 (16)	−0.0090 (14)	−0.011 (2)
C221	0.088 (2)	0.0556 (17)	0.072 (2)	0.0082 (16)	−0.0092 (19)	0.0044 (16)
C222	0.0353 (14)	0.0508 (15)	0.0356 (17)	−0.0079 (12)	−0.0090 (12)	0.0023 (13)
O10	0.0586 (12)	0.0677 (12)	0.0632 (15)	−0.0089 (10)	0.0251 (11)	−0.0067 (10)
O11	0.0702 (13)	0.0505 (11)	0.102 (2)	−0.0034 (10)	0.0021 (13)	0.0043 (11)
O20	0.0656 (13)	0.0721 (12)	0.0595 (15)	−0.0131 (11)	0.0297 (12)	−0.0007 (11)
O21	0.0692 (13)	0.0507 (11)	0.0718 (16)	−0.0028 (9)	−0.0004 (11)	0.0006 (11)

### Geometric parameters (Å, °)

**Table:**

C100—C101	1.405 (4)	C202—C203	1.375 (5)
C100—C105	1.381 (4)	C203—H203	0.9300
C101—H101	0.9300	C204—H204	0.9300
C101—C102	1.373 (4)	C204—C203	1.395 (5)
C102—H102	0.9300	C205—C204	1.386 (4)
C102—C103	1.378 (5)	C206—H206	0.9800
C103—H103	0.9300	C206—C217	1.566 (4)
C104—H104	0.9300	C206—C207	1.529 (4)
C104—C103	1.381 (4)	C206—C205	1.515 (3)
C105—C104	1.402 (3)	C207—H20B	0.9700
C106—H106	0.9800	C207—H20A	0.9700
C106—C117	1.573 (4)	C208—C207	1.521 (4)
C106—C107	1.553 (4)	C208—C209	1.467 (4)
C106—C105	1.508 (3)	C208—C210	1.326 (3)
C107—H10B	0.9700	C210—H210	0.9300
C107—H10A	0.9700	C210—C211	1.450 (4)
C108—C109	1.502 (3)	C211—C212	1.406 (4)
C108—C107	1.498 (4)	C211—C216	1.385 (4)
C108—C110	1.357 (3)	C212—H212	0.9300
C109—C100	1.491 (4)	C212—C213	1.372 (4)
C110—H110	0.9300	C213—H213	0.9300
C110—C111	1.476 (4)	C214—C215	1.383 (4)
C111—C112	1.392 (4)	C214—C213	1.381 (4)
C111—C116	1.389 (4)	C215—H215	0.9300
C112—H112	0.9300	C216—H216	0.9300
C113—H113	0.9300	C216—C215	1.369 (4)
C113—C114	1.375 (4)	C217—C220	1.534 (4)
C113—C112	1.366 (4)	C217—C219	1.526 (4)
C115—H115	0.9300	C217—C218	1.520 (4)
C115—C116	1.381 (4)	C218—H21C	0.9600
C115—C114	1.375 (4)	C218—H21B	0.9600
C116—H116	0.9300	C218—H21A	0.9600
C117—C118	1.532 (4)	C219—H21F	0.9600
C117—C119	1.527 (4)	C219—H21E	0.9600
C117—C120	1.525 (4)	C219—H21D	0.9600
C118—H11F	0.9600	C220—H22C	0.9600
C118—H11E	0.9600	C220—H22B	0.9600
C118—H11D	0.9600	C220—H22A	0.9600
C119—H11C	0.9600	C221—H22F	0.9600
C119—H11B	0.9600	C221—H22E	0.9600
C119—H11A	0.9600	C221—H22D	0.9600
C120—H12C	0.9600	C222—C209	1.462 (4)
C120—H12B	0.9600	C222—C205	1.421 (4)
C120—H12A	0.9600	C222—C201	1.387 (4)
C121—H12F	0.9600	O10—C109	1.226 (3)
C121—H12E	0.9600	O11—C121	1.434 (4)
C121—H12D	0.9600	O11—C114	1.364 (3)
C201—H201	0.9300	O20—C209	1.238 (3)
C201—C202	1.361 (4)	O21—C221	1.430 (3)
C202—H202	0.9300	O21—C214	1.366 (3)
			
C101—C100—C109	118.0 (3)	C202—C201—H201	119.1
C105—C100—C109	121.4 (2)	C202—C201—C222	121.8 (3)
C105—C100—C101	120.7 (2)	C203—C202—H202	120.3
C100—C101—H101	119.7	C201—C202—H202	120.3
C102—C101—H101	119.7	C201—C202—C203	119.4 (3)
C102—C101—C100	120.6 (3)	C204—C203—H203	119.6
C103—C102—H102	120.4	C202—C203—H203	119.6
C101—C102—H102	120.4	C202—C203—C204	120.8 (3)
C101—C102—C103	119.2 (3)	C203—C204—H204	119.9
C104—C103—H103	119.7	C205—C204—H204	119.9
C102—C103—H103	119.7	C205—C204—C203	120.1 (3)
C102—C103—C104	120.5 (3)	C222—C205—C206	119.6 (2)
C105—C104—H104	119.4	C204—C205—C206	121.7 (3)
C103—C104—H104	119.4	C204—C205—C222	118.6 (2)
C103—C104—C105	121.3 (3)	C217—C206—H206	106.3
C104—C105—C106	121.4 (2)	C207—C206—H206	106.3
C100—C105—C106	120.9 (2)	C205—C206—H206	106.3
C100—C105—C104	117.7 (2)	C207—C206—C217	115.1 (2)
C117—C106—H106	105.9	C205—C206—C217	112.84 (19)
C107—C106—H106	105.9	C205—C206—C207	109.3 (2)
C105—C106—H106	105.9	H20A—C207—H20B	107.6
C107—C106—C117	115.1 (2)	C206—C207—H20B	108.6
C105—C106—C117	113.7 (2)	C208—C207—H20B	108.6
C105—C106—C107	109.6 (2)	C206—C207—H20A	108.6
H10A—C107—H10B	107.8	C208—C207—H20A	108.6
C106—C107—H10B	108.9	C208—C207—C206	114.5 (2)
C108—C107—H10B	108.9	C209—C208—C207	115.9 (2)
C106—C107—H10A	108.9	C210—C208—C207	125.9 (2)
C108—C107—H10A	108.9	C210—C208—C209	118.1 (3)
C108—C107—C106	113.2 (2)	C222—C209—C208	119.4 (3)
C107—C108—C109	117.7 (2)	O20—C209—C208	121.0 (3)
C110—C108—C109	115.6 (3)	O20—C209—C222	119.6 (2)
C110—C108—C107	126.8 (2)	C211—C210—H210	113.6
C100—C109—C108	116.9 (2)	C208—C210—H210	113.6
O10—C109—C108	122.2 (2)	C208—C210—C211	132.7 (3)
O10—C109—C100	120.9 (2)	C212—C211—C210	125.4 (2)
C111—C110—H110	115.3	C216—C211—C210	119.1 (2)
C108—C110—H110	115.3	C216—C211—C212	115.5 (2)
C108—C110—C111	129.4 (3)	C211—C212—H212	119.1
C112—C111—C110	125.4 (2)	C213—C212—H212	119.1
C116—C111—C110	117.1 (3)	C213—C212—C211	121.8 (2)
C116—C111—C112	117.4 (3)	C214—C213—H213	119.8
C111—C112—H112	119.7	C212—C213—H213	119.8
C113—C112—H112	119.7	C212—C213—C214	120.4 (2)
C113—C112—C111	120.6 (2)	C213—C214—C215	119.5 (2)
C114—C113—H113	119.4	O21—C214—C215	124.5 (3)
C112—C113—H113	119.4	O21—C214—C213	116.0 (2)
C112—C113—C114	121.2 (3)	C214—C215—H215	120.5
C113—C114—C115	119.6 (3)	C216—C215—H215	120.5
O11—C114—C115	124.4 (3)	C216—C215—C214	119.0 (2)
O11—C114—C113	116.0 (3)	C211—C216—H216	118.1
C116—C115—H115	120.5	C215—C216—H216	118.1
C114—C115—H115	120.5	C215—C216—C211	123.8 (3)
C114—C115—C116	119.0 (2)	C220—C217—C206	112.8 (2)
C111—C116—H116	119.0	C219—C217—C206	109.8 (2)
C115—C116—H116	119.0	C218—C217—C206	110.3 (2)
C115—C116—C111	122.1 (3)	C219—C217—C220	108.6 (3)
C118—C117—C106	109.0 (2)	C218—C217—C220	107.8 (2)
C119—C117—C106	109.6 (2)	C218—C217—C219	107.5 (3)
C120—C117—C106	112.8 (2)	H21B—C218—H21C	109.5
C119—C117—C118	107.4 (3)	H21A—C218—H21C	109.5
C120—C117—C118	108.8 (3)	C217—C218—H21C	109.5
C120—C117—C119	109.1 (3)	H21A—C218—H21B	109.5
H11E—C118—H11F	109.5	C217—C218—H21B	109.5
H11D—C118—H11F	109.5	C217—C218—H21A	109.5
C117—C118—H11F	109.5	H21E—C219—H21F	109.5
H11D—C118—H11E	109.5	H21D—C219—H21F	109.5
C117—C118—H11E	109.5	C217—C219—H21F	109.5
C117—C118—H11D	109.5	H21D—C219—H21E	109.5
H11B—C119—H11C	109.5	C217—C219—H21E	109.5
H11A—C119—H11C	109.5	C217—C219—H21D	109.5
C117—C119—H11C	109.5	H22B—C220—H22C	109.5
H11A—C119—H11B	109.5	H22A—C220—H22C	109.5
C117—C119—H11B	109.5	C217—C220—H22C	109.5
C117—C119—H11A	109.5	H22A—C220—H22B	109.5
H12B—C120—H12C	109.5	C217—C220—H22B	109.5
H12A—C120—H12C	109.5	C217—C220—H22A	109.5
C117—C120—H12C	109.5	H22E—C221—H22F	109.5
H12A—C120—H12B	109.5	H22D—C221—H22F	109.5
C117—C120—H12B	109.5	O21—C221—H22F	109.5
C117—C120—H12A	109.5	H22D—C221—H22E	109.5
H12E—C121—H12F	109.5	O21—C221—H22E	109.5
H12D—C121—H12F	109.5	O21—C221—H22D	109.5
O11—C121—H12F	109.5	C205—C222—C209	120.9 (2)
H12D—C121—H12E	109.5	C201—C222—C209	120.0 (3)
O11—C121—H12E	109.5	C201—C222—C205	119.0 (3)
O11—C121—H12D	109.5	C114—O11—C121	118.6 (2)
C222—C201—H201	119.1	C214—O21—C221	118.1 (2)

### Hydrogen-bond geometry (Å, °)

**Table:**

Cg3 and Cg7 are the centroids of the C111–C116 and C211–C216 rings, respectively.

**Table:**

*D*—H···*A*	*D*—H	H···*A*	*D*···*A*	*D*—H···*A*
C104—H104···O21^i^	0.93	2.53	3.439 (3)	167
C121—H12D···Cg7	0.96	2.92	3.801 (4)	153
C221—H22F···Cg3	0.96	2.86	3.686 (4)	144

Symmetry codes: (i) *x*+1/2, −*y*+1, *z*.
